# Sleep duration and depression among adolescents: Mediation effect of collective integration

**DOI:** 10.3389/fpsyg.2022.1015089

**Published:** 2022-11-28

**Authors:** Min Gao, Xian Li, Chun-Yang Lee, Honghao Ma, Tianmu Chen, Shuoxun Zhang, Yi-Chen Chiang

**Affiliations:** ^1^State Key Laboratory of Molecular Vaccinology and Molecular Diagnostics, School of Public Health, Xiamen University, Xiamen, China; ^2^School of International Business, Xiamen University Tan Kah Kee College, Zhangzhou, China; ^3^Business School, Sichuan University, Chengdu, China

**Keywords:** adolescent, sleep duration, depression, teacher praise, teacher criticism, collective integration

## Abstract

Adolescence is a time of dramatic physical and mental change when adolescents are extremely vulnerable to various mental health problems. Depression and poor sleep duration are increasingly common among adolescents. This study is mainly aimed to verify the important mediating role of collective integration on sleep duration and depression and examine the interrelationship between sleep duration and depression in adolescents longitudinally. The data were obtained from the Wave 1 (in 2013–2014) and Wave 2 (in 2014–2015) longitudinal surveys of China Education Panel Survey (CEPS). The analytic sample in the present study included 8,829 seventh-grade students aged about 14 years (51.50% boys and 48.50% girls). A structural equation modeling (SEM) was used to investigate parent–child/teacher factors affecting adolescent sleep duration and depression, and Monte Carlo resampling with R was employed to confirm the significance of the mediation effects of collective integration. An autoregressive cross-lagged model was employed to analyze the interrelationship between adolescent sleep duration and depression. The findings were as follows. Firstly, collective integration strongly mediated the relationships among academic self-efficacy, parental involvement, teacher praise/criticism, sleep duration, and depression. Secondly, sleep duration and depression were found to have enduring effects and have effects on each other. Thirdly, parental involvement and teacher praise were positively associated with sleep quality and negatively associated with depression. Teacher criticism was negatively associated with sleep quality and positively associated with depression. Compared with teacher praise, teacher criticism has stronger effects on youth sleep duration and depression. In conclusion, improving sleep problems and depression in adolescents as early as possible can stop the persistent and long-term consequences of these problems. Increasing teacher praise, decreasing teacher criticism, and increasing adolescents’ collective integration were effective ways to improve adolescents’ sleep duration and mediate depression.

## Introduction

Adolescence, a period of significant physical, cognitive, emotional, and social change, is a vulnerable time in which individuals are at high risk for psychological health problems (Cyl and Rt, 2021). Sleep disorders are a significant risk factor affecting the physical and mental development of adolescents (Lemola et al., 2015; Liu et al., 2020). Many things prevent teenagers from getting enough sleep, such as academics, social interactions, the internet and even depression and anxiety (Dahl and Lewin, 2002). The American Academy of Pediatrics recognizes sleep deficiency in adolescents has become an important public health problem in modern society (Owens et al., 2014). According to the National Sleep Foundation (NSF), adolescents need 8–10 h of sleep to promote optimal health (Hirshkowitz et al., 2015). A survey of the Parisian adolescent student population confirmed that the average sleep duration of adolescents is well below the recommended values (7 h 14 min) (Stheneur et al., 2019). A cross-sectional population-based study found that more than 87% of U.S. high school students obtain less sleep than they actually need (Patrick and Friedman, 2009). Adequate sleep helps adolescents develop good habits, enhances well-being, improves inattention, and reduces antisocial behavior (Lehto and Uusitalo-Malmivaara, 2014; Widome et al., 2019). Short sleep duration has been shown to be associated with academic performance, mood states, suicidal ideation and suicidal behavior in adolescents (Choquet and Kovess, 1993; Dahl et al., 1996; Liu, 2004; Goodwin and Marusic, 2008; Li et al., 2013; Short and Louca, 2015).

Adolescence is a peak period for the development of depressive disorders (Lewinsohn et al., 1998). An estimated 4.1 million adolescents aged 12 to 17 had at least one major depressive episode. This number represented 17.0% of the U.S. population aged 12 to 17 (National Institute of Mental Health, 2022). Longitudinal studies have estimated that adolescent depression is associated with an increased risk of adult suicide and persistent interpersonal difficulties (Fombonne et al., 2001). Previous studies have established significant negative bidirectional relationships between depression and sleep duration among adolescents (Giannotti et al., 2002; Wang et al., 2021). Poor sleep duration predicts depression in students (Fernandez-Mendoza et al., 2016; Kurtovic and Hnojcik, 2021). Appropriately extended daily sleep duration is essential to prevent adolescent depression (Gangwisch et al., 2010). Depression in adolescents can also significantly affect their sleep duration (Stheneur et al., 2019). Some researchers have found that adolescents with depression experienced more wakefulness, lighter sleep, and more subjective sleep disturbances through meta-analysis of a large volume of literature (Lovato and Gradisar, 2014).

**Figure 1:**
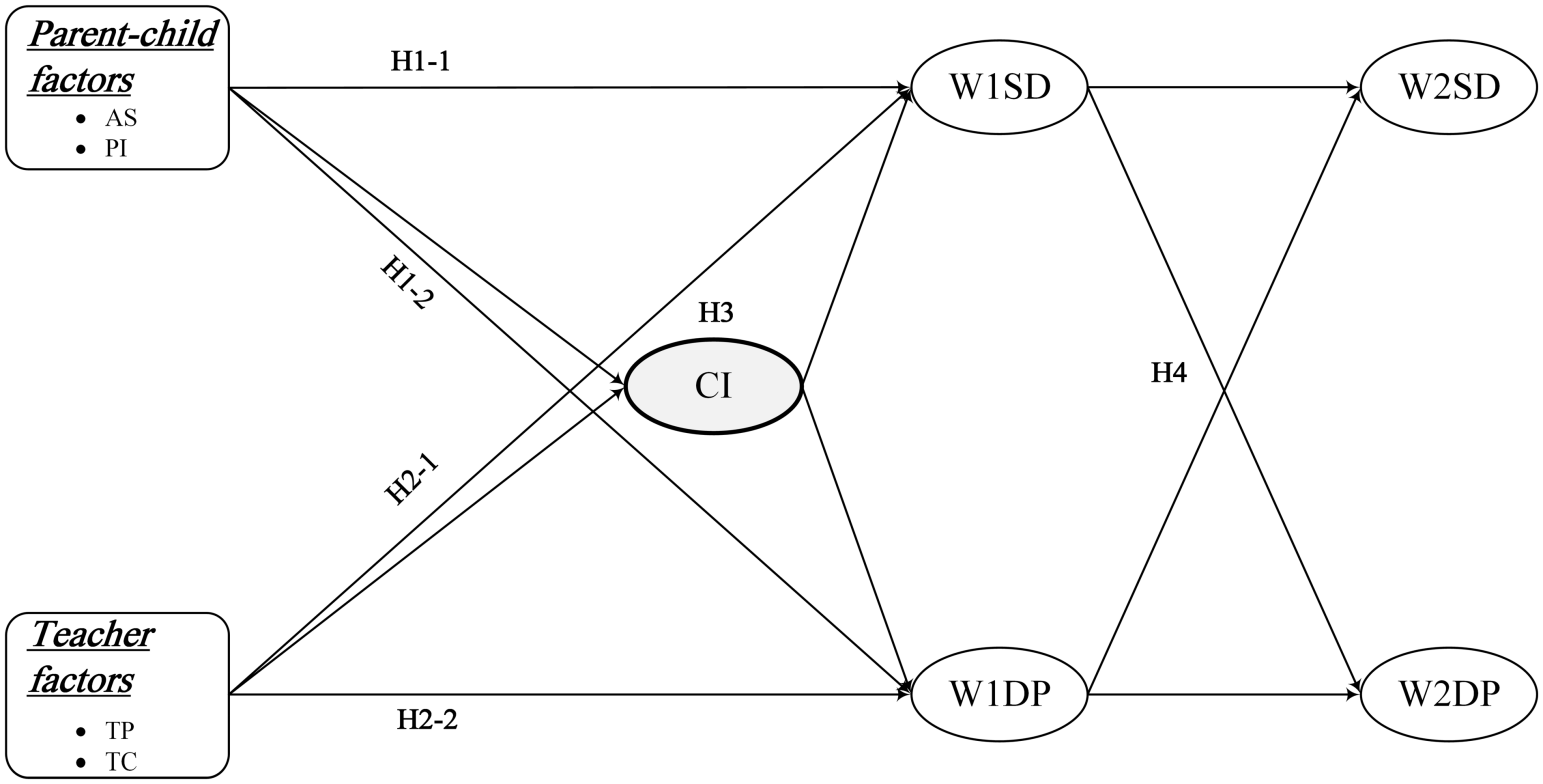
Hypothesized model of the research framework. AS, Academic Self-efficacy; PI, Parental Involvement; TP, Teacher Praise; TC, Teacher Criticism; CI, Collective Integration; W1SD, Sleep Duration in 2013–2014; W1DP, Depression in 2013–2014; W2SD, Sleep Duration in 2014–2015; W2DP, Depression in 2014–2015. Hypotheses were that (H1-1) academic self-efficacy and parental involvement would be positively correlated with sleep duration; (H1-2) academic self-efficacy and parental involvement would be negatively correlated with depression; (H2-1) teacher Praise would be positively correlated with sleep duration, whereas teacher criticism would be negatively correlated with sleep duration; (H2-2) Teacher Praise would be negatively correlated with depression, whereas teacher criticism would be positively correlated with depression; (H3) collective integration would mediate the relationships of parent–child factors and teacher factors with sleep duration and depression; and (H4) there would be a cross effect between sleep duration and depression.

Previous studies have only confirmed the negatively bidirectional relationships between sleep duration and depression in adolescents. Some scholars analyzing sleep duration and depression in adolescents using China Family Panel Studies (CFPS) data have only found that short sleep duration is a risk factor for depression (Zhou et al., 2021). Few studies have confirmed whether sleep deprivation and depression co-occur in adolescence and have a malignant interaction. In addition, the important parent–child and teacher factors affecting sleep duration and depression in adolescents remain unclear. Therefore, this study constructs the structural equation model and the cross-lagged model to be further verified using nationally representative data. We propose the following research hypothesis for the article (Figure 1).

*H4*: there would be a cross effect between sleep duration and depression.

### Theoretical background

Bandura’s social cognitive theory (SCT) is also known as triadic reciprocal determinism, which is a triadic reciprocal causal model that illustrates the forming of personal factors (cognitive, affective and biological events), behavioral patterns and environmental events bi-directionally (Bandura, 1999). Environment is a potential determinant of behavior. Children and adolescents experience major journeys of socialization and development at family and school (Pina et al., 2021). The family is an environment that has a direct impact on the development of children and adolescents (Hosokawa and Katsura, 2020). With the increase of age, students spend a lot of time in school learning and activities, and the school has become the second environment for adolescents to learn and live. In this environment, adolescents’ social relationships mainly involve their parents, teachers, and peers (Tian et al., 2018). Based on the above theory, this study constructs a hypothetical model in which environmental factors (family environment and school environment) are the main variables influencing adolescents’ personal perceptions and thus producing changes in certain behavioral factors to further explore key factors that influence sleep duration and depression in adolescents by using a nationally representative data.

### Parent–child factors that influence adolescent sleep duration and depression

Academic self-efficacy is an individual’s perceived capability to fulfill academic demands, to manage their own learning activities and to fulfill personal, parental, and teachers’ academic expectations (Bandura et al., 1999). In terms of parent–child factors involving individuals and parents, previous study indicated that there was a significant correlation between academic self-efficacy and sleep duration in adolescents (Sook et al., 2020). Specifically, academic self-efficacy improved adolescents’ sleep quality (Bae et al., 2020). Regarding depression, academic self-efficacy had proven that it was negatively associated with depressive symptoms (Scott et al., 2008). Academic self-efficacy moderated depression and reduced the prevalence of depression in Chinese adolescents (Xiang et al., 2020). Similarly, American scholars emphasize academic self-efficacy directly influenced native depression in adolescents (Scott et al., 2008).

Although adolescents begin to individuate from their families and increase the scope and intensity with peers, they still need the support and approval of their parents (Chen et al., 2006). Parental involvement was found to be associated with sleep functioning in adolescents (Roblyer and Grzywacz, 2015). When the level of parental involvement was lower, adolescents had poorer sleep quality and higher levels of psychological distress (Cousins et al., 2007). A qualitative study in England also highlighted the importance of parental involvement in ensuring adolescents’ healthy sleep (Godsell and White, 2019). In addition, parental involvement was closely related to the cognitive, psychological and behavioral development of adolescents (Wu and Li, 2021). Parental involvement has been found to improve adolescents’ emotional functioning (Wang and Sheikh-Khalil, 2014). Higher degree of parental involvement and a strong bond between youth and their parents have been found to be associated with a reduced likelihood of youth depression and loneliness (Fröjd et al., 2007). Based on the above, we propose the following research hypotheses (Figure 1).

***H1-1***: academic self-efficacy and parental involvement would be positively correlated with sleep duration.

***H1-2***: academic self-efficacy and parental involvement would be negatively correlated with depression.

### Teacher factors that influence adolescent sleep duration and depression

In terms of teacher factors, teacher praise and criticism are important teacher factors affecting adolescent sleep duration and depression. Positive teacher teaching strategies like teachers praising students contribute to student well-being (Wang and Kuo, 2019). Specifically, teacher praise has a significant positive impact on adolescent mental health (Palsdottir et al., 2012). Teachers use praise can increase adolescent self-esteem, and high self-esteem is a protective factor against depression and contributes to socially desirable behaviors and self-improvement (Baumeister et al., 2003). When teachers use “angry blame” and “ridicule,” students have the least emotional pleasure (Wei, 2015). Adolescents are vulnerable to the negative effects of being criticized by their teachers. Teachers use negative remarks frequently create a negative classroom climate and evoke feelings of upset, anxiety, and depression among students (Cadima et al., 2015). Particularly, students who receive teacher criticism are more likely to experience higher levels of depression than their peers (Thompson et al., 2021). This negative emotional experience caused by teacher criticism affects the sleep quality of teenagers and shortens their sleep time (Delaruelle et al., 2021). According to Bandura’s social cognition theory, teacher factors in the school environment are the key influencing factors of students’ sleep and emotional experience, so it is necessary to discuss the influence of teacher praise and criticism on students. The hypotheses are established as follows (Figure 1).

***H2-1***: Teacher Praise would be positively correlated with sleep duration, whereas teacher criticism would be negatively correlated with sleep duration.

***H2-2***: Teacher Praise would be negatively correlated with depression, whereas teacher criticism would be positively correlated with depression.

### Collective integration has mediating effects on youngsters’ sleep duration and depression

Firstly, the need for human communication increases significantly during adolescence (Gogitsaeva et al., 2019). Adolescents actively seek out peers and groups and approval and inclusion. Teenagers’ poor daily interpersonal interactions shape substandard sleep quality and shorter sleep duration (Chiang et al., 2019). Meanwhile, increased feelings of loneliness which means a loss of collective integration predicts greater insomnia and depressive symptoms (Yen et al., 2008; Chu et al., 2019). The study found that negative interpersonal interaction with peers generates higher levels of depression and more harmful emotions (Zimmer-Gembeck et al., 2007). Adolescents’ popularity in the collective made an independent contribution to predicting depressive symptoms (Oldenburg and Kerns, 1997).

Secondly, in terms of parent–child factors, lower academic self-efficacy in adolescents is associated with higher social anxiety (Levpuscek and Berce, 2012). Higher social anxiety in adolescents implies lower collective integration. The higher adolescents’ academic self-efficacy is, the more integrated they are into the school group and the stronger their sense of belonging is (Arslan and Duru, 2017). Furthermore, a study on the collective integration of Chinese international students showed that the more parents were involved, the less lonely the students felt and the easier it was for them to integrate into the group (Su et al., 2021).

Thirdly, in terms of teacher factors, on the one hand, teacher praise plays a key role in adolescent development by providing emotional support that helps adolescents build and maintain close relationships with family and peers and thus helps them better integrate into the group (Heilat and Seifert, 2019). On the other hand, too much teacher criticism hinders students’ sense of security and self-esteem, which further hampers teenagers’ collective integration behaviors, such as participating in group activities or communicating with peers and classmates (Cadima et al., 2015). Verbal praise from teachers, rather than reprimands, helps create a predictable, friendly, and safe environment, which helps strengthen students’ collective integration (Wilson et al., 2007; Spilt et al., 2016).

Guided by Bandura’s social cognitive theory, both parent–child and teacher factors can have an impact on adolescents’ sleep duration and depression. Yet few studies have explored the important mediating factors involved. Hence, this study tries to explore the mediating effect of adolescent group inclusion on parent–child factors/teacher factors and sleep duration/depression. The hypotheses are established as follows (Figure 1).

***H3***: Collective integration would mediate the relationships of parent-child factors and teacher factors with sleep duration and depression

## Materials and methods

### Data

Data were obtained from two waves of the China Education Panel Survey (CEPS) which was a nationally representative survey of lower-secondary school students (Grades 7–9) conducted from 2013 to 2015 by the National Survey Research Center at Renmin University of China. In the baseline academic year of 2013–2014, the CEPS recruited a nationally representative sample of junior high school students by multistage probability proportional to the size sampling method. In this project, participants were selected from 112 junior high schools in 28 county-level districts in mainland China. At the first stage, 28 counties/districts were chosen as primary sampling units from 2870 counties/districts with probability proportional to size (PPS). At the second stage, four schools were chosen from each county/district. At the third stage, two classes were randomly selected from the seventh grade, and two classes were randomly selected from the ninth grade in each school. At the fourth stage, all the students from the 438 classes, their parents, and the school personnel from each selected classroom were surveyed as participants. Four rounds of follow-up surveys were conducted from 2014 to 2018, one in each academic year, but currently only two rounds of data (2013–2014 and 2014–2015) are publicly available. Our analysis is therefore based on these two available rounds of data. In order to exclude the possible influence of ninth graders’ senior high school entrance examination and promotion to high school on the results during the two waves of follow-up, only 10,279 14-year-old seventh graders were selected for this study. Finally, a total of 8829 students who were successfully followed up for both sleep duration and depression between the two waves were included in the data analysis (valid sample rate: 85.90%).

### Procedure

The study population for the 2013–2014 baseline survey was 10,279 seventh grade students, and all students’ questionnaires should be filled out centrally in the school classroom within the specified time, completed and collected in the same classroom. Students who were absent from school for special reasons should take their student questionnaires home to fill them out and collect them in time. In each classroom, the partner should ensure that at least 2 trained surveyors are arranged to complete the on-site survey while cooperating with each other. The majority of 7th grade students in the 2013–2014 baseline survey have normally moved on to 8th in 2014–2015 follow-up survey. For students who have not been placed in a class after moving, they should fill out the questionnaire in the school classroom within the specified time, complete it in the same classroom and collect it all. For students who have been divided into different classes, classroom teachers should be asked to confirm the classes of these students and arrange a suitable time to gather the students together to fill in the questionnaire. For students who were absent from school due to special reasons should take the student questionnaires home to fill out and collect them in time. Before the survey started, the project team actively communicated with the research subjects to clarify the purpose of the study and the research process to them. Finally, participants’ written informed consent was obtained by the National Survey Research Center of Renmin University, PR China, and human ethics approval was obtained from Renmin University of China.

The pilot survey of China Education Panel Survey (CEPS) was conducted in 2012 to verify the rationality and feasibility of the study protocol. It was conducted using multi-stage cluster sampling, and the CEPS project team visited the target schools to organize students, parents, and teachers to complete and return the questionnaires on the spot, with a 100% response rate. The pilot survey involved a total of 2306 adolescents, including 1219 boys (52.86%) and 1087 girls.

### Measures

#### Sleep duration

Sleep duration was measured by asking students in wave 1 and wave 2, “How much time on average do you sleep every night.”

#### Depression

The following five items derived from a short version of the Center for Epidemiologic Studies Depression Scale were used to assess students’ depression. “Do you have the feelings below in the last 7 days? Feel blue/Depressed/unhappy/Not enjoying life/Sad.” Responses ranged from “1 = never” to “5 = always.” Depression was measured by asking students in wave 1 and wave 2. The scores for each of the five items were summed to give an overall total score that ranged from 5 to 25. A high score corresponds to a high level of depression. This measurement was used in prior studies with Chinese adolescents and shown to have good reliability (Jiang et al., 2017; Jiang and Dong, 2020). In the present sample, the depression scale had a Cronbach’s alpha of 0.85, indicating good reliability.

#### Academic self-efficacy

Academic self-efficacy scales in this study were adapted from the Aggressiveness Scale (Cai and Wu, 2016). Previous study has also verified its application in the CEPS survey study (Huang, 2018). Students’ academic self-efficacy in grade 7 was measured by following four items. And it was investigated in Wave 2, in which students were asked to recall their academic self-efficacy in seventh grade. “How much do you agree with each of the following statements about your experiences in GRADE 7? I would try my best to go to school even if I was not feeling very well or I had other reasons to stay at home/ I would try my best to finish even the homework I dislike/I would try my best to finish my homework, even if it would take me quite a long time/I would persist in my interests and hobbies.” Strongly disagree = 1, somewhat disagree = 2, somewhat agree = 3, and strongly agree = 4. The overall total scores ranged from 4 to 16. A higher average score indicated a higher level of academic self-efficacy. In the present sample, the academic self-efficacy scale had a Cronbach’s alpha of 0.80, indicating good reliability.

#### Parental involvement

Parental involvement covers the parenting of children as well as family activities (Yang, 2019). Parental involvement was measured by responses to eight questions in wave 1: “How often do your father/mother discuss the following with you? things happened at school/the relationship between you and your friends/the relationship between you and your teachers/your worries and troubles.” Students’ parental involvement scored on a three-point scale (1 = never, 2 = sometimes, 3 = often). A higher average score indicated a higher level of parental involvement. In this study, the parental involvement scale had a Cronbach’s alpha of 0.83, indicating good reliability.

#### Teacher praise

The CEPS uses four self-assessment items to examine students’ perceived teacher praise (Jiang et al., 2017). Teacher praise was measured by responses to four prompts in wave 1: “My mathematics teacher always praises me/My Chinese teacher always praises me/My English teacher always praises me/My homeroom teacher always praises me.” Each item was scored from strongly disagree = 1, somewhat disagree = 2, somewhat agree = 3, to strongly agree = 4. A higher value indicating a higher level of teacher praise. The Cronbach’s alpha for these items was 0.85, indicating good reliability.

#### Teacher criticism

Teacher criticism was measured by the following two items in wave 1: “My parents always receive criticism on me from my teacher”; and “My homeroom teacher always criticizes me.” Each item was scored from strongly disagree = 1, somewhat disagree = 2, somewhat agree = 3, and strongly agree = 4. A higher value indicating a higher level of teacher criticism. The Cronbach’s alpha for these items was 0.60, indicating acceptable reliability.

#### Mediating variable

In this study, collective integration was hypothesized to play a mediating role between dependent and independent variables. The CEPS explores adolescents’ collective integration from the perspective of collective belonging and identity, together with participation in school activities (Huang, 2018). Collective integration was measured by the following three items in wave 1: “Most of my classmates are nice to me”; “I often take part in school/class activities”; and “I feel close to people in this school.” Each item was followed by a 4-point scale ranging from “1 = strongly disagree” to “4 = strongly agree.” A higher score indicated a higher level of collective integration. The Cronbach’s alpha for these items was 0.71, indicating acceptable reliability.

We conducted confirmatory factor analysis (CFA) for the instruments used to measure depression, academic self-efficacy, parental involvement, and teacher praise. The goodness-of-fit index of depression was as follows: (1) chi-square/df = 39.74; (2) RMSEA = 0.065; (3) GFI = 1.00; (4) AGFI = 0.99; (5) TLI = 0.96; and (6) CFI = 0.98. The goodness-of-fit index of academic self-efficacy was as follows: (1) chi-square/df = 59.94; (2) RMSEA = 0.079; (3) GFI = 1.00; (4) AGFI = 0.99; (5) TLI = 0.98; and (6) CFI = 0.99. The goodness-of-fit index of parental involvement was as follows: (1) chi-square/df = 217.68; (2) RMSEA = 0.159; (3) GFI = 0.96; (4) AGFI = 0.93; (5) TLI = 0.86; and (6) CFI = 0.90. The goodness-of-fit index of teacher praise was as follows: (1) chi-square/df = 97.72; (2) RMSEA = 0.102; (3) GFI = 1.00; (4) AGFI = 0.98; (5) TLI = 1.00; and (6) CFI = 0.97. These outcomes indicate that all the instruments have good validity.

#### Control variables

Several control variables were considered in this study: sex (female = 1, male = 2); live with parents (Yes or No) (live with both parents = 1, only one or neither = 2); and parental highest educational level (primary school or below = 1, junior high school = 2, secondary school/technical school/vocational high school/senior high school = 3, junior college/undergraduate college/graduate and above = 4).

### Data analysis

Statistical analyses were performed using SAS version 9.4 (Copyright © SAS Institute Inc., SAS Campus Drive, Cary, North Carolina 27513, United States. All rights reserved), LISREL version 8.80 (Copyright 2006, Scientific Software International Inc., All rights reserved), and R version 4.0.2 (R Foundation, Vienna, Austria). The distributions of the control, independent, mediating, and dependent variables are presented as the frequency distribution and percentages, means and standard deviations by SAS. To examine whether our hypotheses could be empirically supported, structural equation modeling (SEM) was performed with LISREL to analyze our data in two waves. In addition, the estimated indirect effects (IEs) in the output file from LISREL and Monte Carlo resampling with R were used to confirm the significance of the IEs.

## Results

### Participant characteristics

Descriptive statistics of the sample and mean of variables are presented in Table 1. A total of 8,829 students were selected, including 4,550 boys (51.50%) and 4,279 girls (48.50%). The sex ratio was approximately 1.06:1.

**Table 1:** Demographic characteristics of participants and descriptive statistics of key variables. (*N* = 8829).

Variable	ME	*SD*	*n* ^#^	%
Control variables				
Sex				
Female			4,279	48.50
Male			4,550	51.50
Live with parents (Yes or No)				
Both parents			6,825	77.30
Only one or neither			2,004	22.70
Parental highest education level				
≤Primary school degree			682	7.70
Junior high school degree			3,730	42.20
≤Senior high school degree			2,622	29.70
≥Junior college degree			1,795	20.30
Independent variables				
Academic self-efficacy [1–4]	3.20	0.68		
Parental involvement [1–3]	2.07	0.50		
Teacher praise [1–4]	2.58	0.76		
Teacher criticism [1–4]	1.50	0.65		
Dependent variables				
W1 sleep duration (hours)	8.35	1.09		
W1 depression [1–5]	1.99	0.79		
W2 sleep duration (hours)	8.00	1.07		
W2 depression [1–5]	2.16	0.90		
Mediating variables				
Collective integration [1–4]	3.03	0.73		

ME, Mean; SD, Standard deviation; W1 sleep duration, sleep duration in 2013–2014; W1 depression, depression in 2013–2014; W2 sleep duration, sleep duration in 2014–2015; W2 depression, depression in 2014–2015.

^#^The total number < *n* = 8829 due to missing.

[]: The range of a single item.

### Direct/indirect and mediation effect analysis

**Figure 2:**
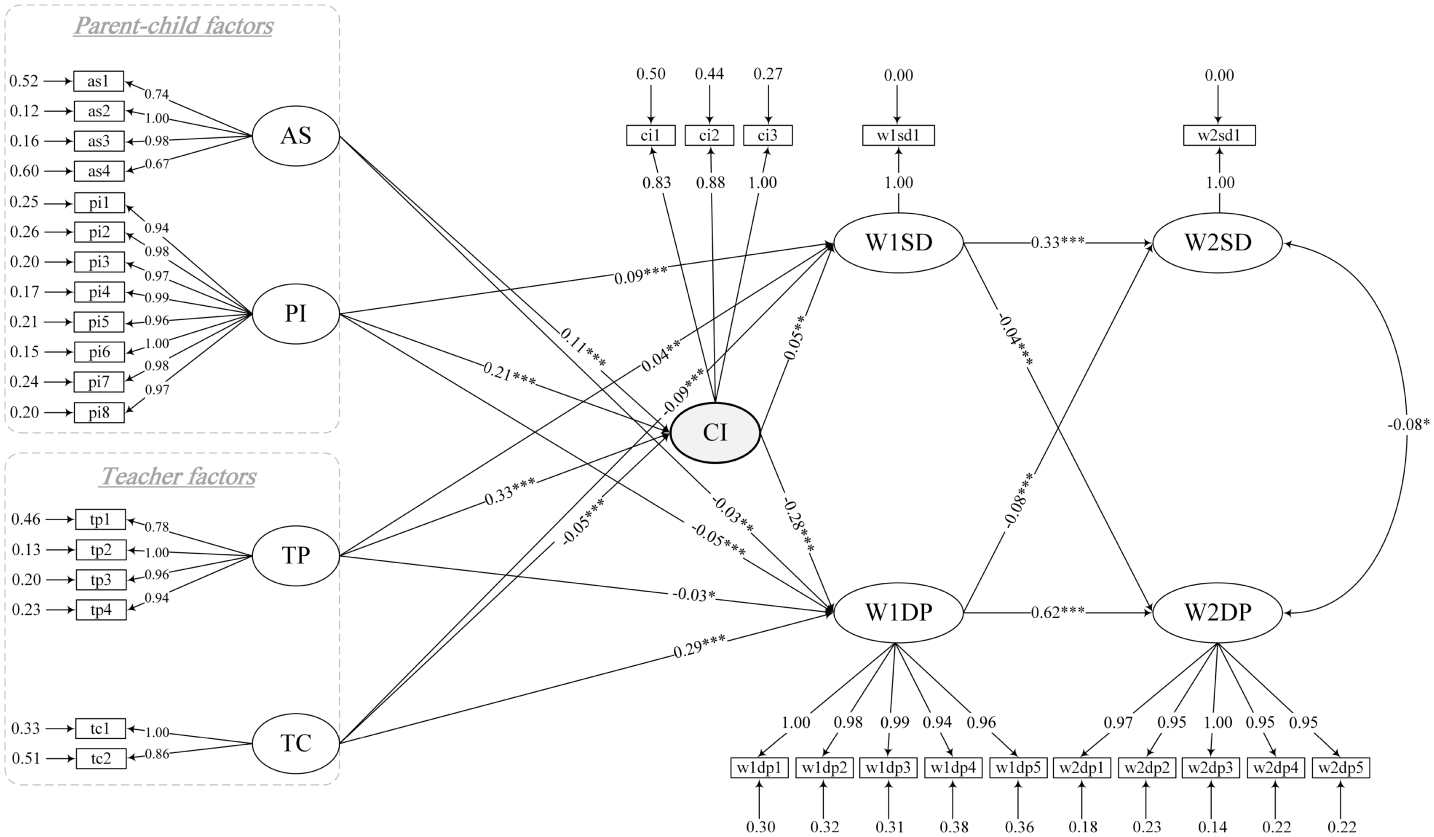
Model diagram of the mediating effect of collective integration on sleep duration and depression among adolescents. In this model, sex, live with parents (Yes or No), and parental highest education level are taken as control variables. AS, Academic self-efficacy; PI, Parental involvement; TP, Teacher praise; TC, Teacher criticism; CI, Collective integration; W1SD, Sleep duration in2013-2014; W1DP, Depression in 2013–2014; W2SD, Sleep duration in 2014–2015; W2DP, Depression in 2014–2015. *p < 0.05, **p < 0.01, ***p < 0.001.

Based on the present model, collective integration was added to the structural equation model as a mediating variable in the direct path from parent–child factors (academic self-efficacy/parental involvement) and teacher factors (teacher praise/teacher criticism) to adolescents’ sleep duration and depression after controlling for sex, living with parents (Yes or No), and parental highest level of education (Figure 2). According to the smallest and nonsignificant t values, the final model was obtained by deleting LWP → W1SD and AS → W1SD. The final model showed improved overall fit compared to the initial model: Chi-Square/DF = 13.33, RMSEA = 0.040, NFI = 0.93, TLI = 0.93, CFI = 0.94, IFI = 0.94, CN = 647.85, GFI = 0.98, and AGFI = 0.98 (Table 2). The final model is shown in Figure 2.

**Table 2:** Measures of goodness-of-fit for the competition models.

	Chi-square/DF	RMSEA	NFI	TLI	CFI	IFI	CN	GFI	AGFI
Initial model	7356.30/550	0.041	0.93	0.93	0.94	0.94	645.71	0.98	0.98
Delete LWP → W1SD	7356.51/551	0.041	0.93	0.93	0.94	0.94	646.79	0.98	0.98
^#^Delete AS → W1SD	7356.85/552	0.040	0.93	0.93	0.94	0.94	647.85	0.98	0.98

LWP, live with parents (Yes or No); W1SD, sleep duration in 2013–2014; AS, academic self-efficacy.

^#^The goodness-of-fit of the Final model.

The pathways from parent–child and teacher factors for sleep duration and depression are presented in Table 3. There was significant direct positive effects of parental involvement and teacher praise on students’ sleep duration (*β* = 0.09, *p* < 0.001; *β* = 0.04, *p* < 0.01). There was a direct negative effects of teacher criticism on students’ sleep duration (*β* = −0.09, *p* < 0.001). The above results support H1-1 and H2-1. The direct effects of academic self-efficacy, parental involvement and teacher praise on students’ depression were negative (*β* = −0.03, *p* < 0.01; *β* = −0.05, *p* < 0.001; *β* = −0.03, *p* < 0.05). The direct effect of teacher criticism on students’ depression was significantly positive (*β* = 0.29, *p* < 0.001).The above results support H1-2 and H2-2. Collective integration (*β* = 0.05, *p* < 0.01) was significantly positively correlated with sleep duration in 2013–2014; in contrast, it (*β* = −0.28, *p* < 0.001) was significantly negatively correlated with depression in 2013–2014.

**Table 3:** Descriptive statistics for the direct effect, indirect effect and total effect of the critical Parent–child/teacher factors, and mediating variable on adolescents sleep duration and depression.

	W1SD			W1DP			W2SD			W2DP		
	DE	IE	TE^a^	DE	IE	TE^a^	DE	IE	TE^a^	DE	IE	TE^a^
***Parent–child***												
AS	––	0.006^**^	0.006^**^	−0.030^**^	−0.030^**^	−0.060^***^	––	0.010^**^	0.010^**^	––	−0.040^***^	−0.040^***^
PI	0.090^***^	0.010^*^	0.100^***^	−0.050^***^	−0.060^***^	−0.110^***^	––	0.040^***^	0.040^***^	––	−0.070^***^	−0.070^***^
***Teacher***												
TP	0.040^**^	0.020^**^	0.060^***^	−0.030^*^	−0.100^***^	−0.130^***^	––	0.030^***^	0.030^***^	––	−0.080^***^	−0.080^***^
TC	−0.090^***^	−0.003^*^	−0.093^***^	0.290^***^	0.010^**^	0.300^***^	––	−0.060^***^	−0.060^***^	––	0.190^***^	0.190^***^
***Mediating variables***												
CI	0.050^**^	––	0.050^**^	−0.280^***^	––	−0.280^***^	––	0.040^***^	0.040^***^	––	−0.170^***^	−0.170^***^

AS, Academic self-efficacy; PI parental involvement, TP Teacher praise, TC Teacher criticism, CI Collective integration, W1SD Sleep duration in 2013–2014, W1DP Depression in 2013–2014, W2SD Sleep duration in 2014–2015, W2DP Depression in 2014–2015, DE Direct effect, IE Indirect effect, TE Total effect; *p < 0.05, **p < 0.01, ***p < 0.001.

^a^The total effect refers to the value of the output result of the model, and part of it is slightly different from the total value of the direct and indirect effects due to rounding.

The overall direct effect, indirect effect and total effect of independent variables on adolescent sleep duration and depression were calculated by LISREL and are shown in Table 3. The table indicates that the indirect effect of academic self-efficacy on sleep duration in 2013–2014 was 0.006 [0.11*0.05]; the indirect effect of parental involvement on sleep duration in 2013–2014 was 0.01 [0.21*0.05]; the indirect effect of teacher praise on sleep duration in 2013–2014 was 0.02 [0.33*0.05]; and the indirect effect of teacher criticism on sleep duration in 2013–2014 was −0.003 [(−0.05)*0.05]. The table also indicates that the indirect effect of academic self-efficacy on depression in 2013–2014 was −0.03 [0.11*(−0.28)]; the indirect effect of parental involvement on depression in 2013–2014 was −0.06 [0.21*(−0.28)]; the indirect effect of teacher praise on depression in 2013–2014 was −0.10 [0.33*(−0.28)]; and the indirect effect of teacher criticism on depression emotion in 2013–2014 was 0.01 [(−0.05)*(−0.28)]. In summary, collective integration had significant mediating effects on the relationships between independent and dependent variables (sleep duration/depression). Thus, for the most part, H3 are supported.

To further verify the mediating effects of collective integration, Monte Carlo resampling was used to construct the 95% Confidence Intervals (CIs). Specifically, a program was written in R to construct 95% CIs for the IEs based on 20,000 resamples (Preacher, 2012). According to the results (Table 4), the 95% CI for all indirect effects did not include zero. Therefore, H4 were further supported.

**Table 4:** Tests of indirect effects of collective integration in the hypothesized model by Monte Carlo approach of resampling (total sample, *n* = 8829).

Path	Indirect effect^a^	95%CI^a^
AS → CI → W1SD	0.009	[0.0017, 0.0095]
PI → CI → W1SD	0.010	[0.0034, 0.0176]
TP → CI → W1SD	0.020	[0.0054, 0.0275]
TC → CI → W1SD	−0.003	[−0.0053, −0.0004]
AS → CI → W1DP	−0.030	[−0.0389, −0.0233]
PI → CI → W1DP	−0.060	[−0.0692, −0.0493]
TP → CI → W1DP	−0.100	[−0.1072, −0.0786]
TC → CI → W1DP	0.010	[0.0040, 0.0239]

AS, Academic self-efficacy; PI, Parental involvement; TP, Teacher praise; TC, Teacher criticism; CI, Collective integration; W1SD, Sleep duration in 2013–2014; W1DP, Depression in 2013–2014.

^a^The total effect refers to the value of the output result of the model, and part of it is slightly different from the total value of the direct and indirect effects due to rounding.

This study evaluated a cross-lagged model in order to reveal a lasting cross-sectional relationship between sleep duration and depression in adolescents. W1SD was positively correlated with W2SD (*β* = 0.33, *p* < 0.001), and W1DP was positively correlated with W2DP (*β* = 0.62, *p* < 0.001). These results indicated that students’ sleep duration and depression have enduring effects. W1SD was negatively correlated with W2DP (*β* = −0.04, *p* < 0.001), and W1DP was negatively correlated with W2SD (*β* = −0.08, *p* < 0.001), indicating that sleep duration and depression may affect each other persistently; thus, H4 was supported. In this cross-lagged model, first, the synchronous coefficient between sleep duration in 2014–2015 and depression in 2014–2015 was −0.08 (*p* < 0.001). Second, stationarity coefficients were 0.33 (*p* < 0.001) between sleep duration in 2013–2014 and sleep duration in 2014–2015 and 0.62 (*p* < 0.001) between depression in 2013–2014 and depression in 2014–2015. Third, cross-lagged coefficients were − 0.04 (*p* < 0.001) between sleep duration in 2013–2014 and depression in 2014–2015 and − 0.08 (*p* < 0.001) between depression in 2013–2014 and sleep duration in 2014–2015.

Furthermore, in order to explore the causality between sleep duration and depression, we used students without excessive depression/with normal sleep duration in the 2013–2014 baseline survey as study subjects to construct sub-model 1 and sub-model 2, respectively, in Supplementary materials. Firstly, the depression scale consisted five questions and responses ranging from “1 = never” to “5 = always.” The scores were summed to give an overall total score that ranged from 5 to 25. Strictly speaking, students with a total score of no less than 20 were considered to have excessive depressive emotion. After exclusion, the remaining 8738 individuals were used as study subjects in sub-model 1 (Figure 1 in Supplementary material). The final sub-model 1 using adolescents without depression showed improved overall fit: Chi-Square/DF = 13.03, RMSEA = 0.040, NFI = 0.93, TLI = 0.93, CFI = 0.94, IFI = 0.94, CN = 655.91, GFI = 0.98, and AGFI = 0.98 (Table 1 in Supplementary material). Secondly, according to the National Sleep Foundation (NSF), adolescents need to sleep no less than 8 h per day. And in China, middle school students are required to take a two-hour lunch break every day, so they should sleep at least 6 h per night. Based on this, the study individuals with less than 6 h of sleep per night were excluded, and the remaining 8773 individuals were used as the study subjects in sub-model 2 (Figure 2 in Supplementary material). The final sub-model 2 using adolescents with normal sleep duration showed improved overall fit: Chi-Square/DF = 13.10, RMSEA = 0.040, NFI = 0.93, TLI = 0.93, CFI = 0.94, IFI = 0.94, CN = 655.29, GFI = 0.98, and AGFI = 0.98 (Table 2 in Supplementary material). The results showed that sleep duration and depression in adolescents were causally related to each other. Among adolescents without excessive depressive emotion, sleep duration in the wave 1 can directly and negatively affect depression in the second year (*β* = −0. 03, *p* < 0. 001); meanwhile sleep duration in the wave 1 can directly have a greater positive impact on sleep duration in the second year (*β* = 0. 39, *p* < 0. 001). And there was a correlation between sleep duration and depression in the same year, with synchronous coefficients of −0. 09, *p* < 0. 001 (Figure 1 in Supplementary material). In adolescents with normal sleep duration, depression in the wave 1 could directly affect the sleep duration in the wave 2 negatively (*β* = −0. 08, *p* < 0. 001); depression in the wave 1 largely had a greater positive effect on depression in the wave 2 (*β* = 0. 61, *p* < 0. 001). And there was a correlation between sleep duration and depression in the same year, with synchronous coefficients of −0.08, *p* < 0.001 (Figure 2 in Supplementary material).

## Discussion

In this study, a mediating model was used to test the mediating effects of collective integration and the direct effect of some parent–child and teacher factors on students’ sleep duration and depression. Additionally, the results showed that sleep duration and depression may be cross affected by each other and have enduring effects. On this basis, some suggestions are given to protect students from poor sleep duration and depression.

### Sleep duration and depression may affect each other and have enduring effects

Previous cross-sectional studies have confirmed a negative association between sleep duration and depression in adolescents (Yang and Cha, 2018; Stheneur et al., 2019). However, the continuous interaction between sleep duration and depression has rarely been explored. Our cross-lagged model in this study further showed that sleep duration and depression among adolescents may interact with each other and have enduring effects. First, adolescents’ sleep duration and depressive had a positive auto-regressive effect, respectively. Second, adolescents’ sleep duration in the previous year was a causal predictor of depression in the next year. Similarly, adolescents’ depression in the previous year was a causal predictor of sleep duration in the next year. Third, there was a negative correlation between sleep duration and depression in the same year. Besides, innovative findings of this study were the lasting effects and interactions produced by depression in adolescents are stronger than those produced by sleep duration. Therefore, only early prevention and treatment of depression and improvement in sleep quality can effectively stop further deterioration of the situation. In the context of the post-epidemic era with rapid development of digital technology, the emotional management of adolescents should be paid more attention to (Ribeiro-Navarrete et al., 2021; Saura et al., 2022). As Stheneur et al. (2019) suggested, qualitative and quantitative assessments of sleep should be included in adolescents’ physical examination, and a psychological assessment scale for depression should be administered for early detection, prevention and treatment.

### Collective integration plays a crucial mediating role in adolescents’ sleep duration and depression

A study found that adolescents with external manifestations such as mood disorders or social isolation were often had symptoms of insomnia or short sleep duration and depression (Fernandez-Mendoza et al., 2016). Social isolation may be a unique risk factor for depression in early adolescence (Richardson et al., 2019). Isolated adolescents do not get along well with their peers at school, which is what we call poor collective integration. Higher isolation was associated with higher frequency of difficulties sleeping, greater sleep disturbance and more instances of feeling tired in the morning (Eccles et al., 2020). This study has found that collective integration plays an important mediating role in the relationship between parent–child/teacher factors and sleep duration/depression among adolescents. The more well integrated adolescents are, the longer they sleep. Further, the less able they are to integrate into a group and get along well with classmates, the stronger the depression. Additionally, the effect of collective integration on teenagers’ depression is greater than that of teenagers’ sleep duration. Thus, enhanced collective integration may be an effective modifiable protective factor in the prevention of adolescent depression. Enhanced collective integration is also a highly effective approach in ensuring that adolescents receive adequate sleep. The results of this study also showed that academic self-efficacy, parental involvement, and teacher praise/criticism all had direct effects on adolescents’ collective integration. In particular, parental involvement and teacher praise had the strongest effects on collective integration. Therefore, we suggest that parents should actively communicate with their children, express their concerns and interests in their school life, be understanding and provide advice when adolescents encounter difficulties and challenges. Parents should be role models and encourage youth to leave the house and integrate courageously into peer groups and society. Additionally, we recommend that teachers adopt an encouraging approach to adolescents and reduce the criticism that undermines their self-confidence, especially in front of the whole class. Teachers should be kind and use words of praise to encourage high self-esteem, rather than harsh public accusations and comparisons that discourage self-esteem.

### Compared with teacher praise, teacher criticism has stronger negative effects on youth sleep duration and depression

Teachers guide students and are the most frequent, direct, and authoritative role models for them. Teacher praise and reprimands can be tools to potentially influence student behavior and classroom behavior (Sabey et al., 2019) Teacher praise and criticism are very important and plastic classroom management strategies that play an important role in behavioral intervention and emotion regulation (Spilt et al., 2016; Wills et al., 2021; Zoromski et al., 2021). Compliments can significantly improve interpersonal interactions for adolescents, even for those who lack social skills, and praise can be a good motivator for them to integrate and interact with others (Berner et al., 2001). Most adolescents report that their school life is happiest and most relaxed when they are on good terms with their friends and when they receive praise from their teachers (Wills et al., 2021). We found that both teacher praise and criticism produced direct effects on adolescents’ sleep duration and depression. However, teacher criticism produced a stronger negative effect than teacher praise. Smith et al. (2009) also found that self-reported depression symptoms in late adolescence are associated with teacher criticism. Numerous studies have examined the association between parental praise and youth depression (Butterfield et al., 2021; Gadassi-Polack et al., 2021). However, few studies have been conducted to verify the effect of teacher praise on adolescent depression. This study fills that gap. Teachers who guide adolescent development should engage in positive, equal communication with students. The simplest and easiest approach is to increase teacher praise and decrease teacher criticism. It is especially important to note that teacher criticism can more stronger negatively contribute to adolescent mental health, such as sleep problems and depression. Furthermore, teachers’ critical approach to education is more likely to provoke rebellion in adolescent students, which is counterproductive.

### Major manifestations of academic self-efficacy and teacher praise/criticism in the eyes of adolescents

According to λ values (λ > 0.85) in the structural equation model, we found that students’ academic self-efficacy was expressed in response to two main prompts: (1) I would try my best to finish my homework, even if it would take me quite a long time (λ = 0.98); (2) I would try my best to finish even the homework I dislike (λ = 1.00). The results of this study found that increasing adolescents’ academic self-efficacy must first increase adolescents’ self-motivation and incentive to complete difficult or disagreeable homework. Moreover, regarding teacher praise, the main influence of teacher praise on students comes from Chinese teacher praise (λ = 1.00), English teacher praise (λ = 0.96), and homeroom teacher praise (λ = 0.94). Teachers’ criticism has a negative impact on students mainly because teachers share this criticism with parents (λ = 1.00), followed by the direct impact of the criticism on the students (λ = 0.86). These findings can be applied to improve the collective integration, sleep quality or duration, and depression of adolescents. On the one hand, positive praise from Chinese teachers, English teachers, and homeroom teachers is more effective. On the other hand, when students make mistakes, teachers’ criticism of students should be accomplished face to face with students instead of informing students’ parents in order to protecting the students’ mental health, except in the event of serious problems.

### Theoretical implications

In Bandura’s social cognitive theory (Bandura, 1986), there are interactions between personal factors (e.g., cognitions, feelings, skills), behavioral factors (e.g., strategy use, help-seeking actions), and environmental factors (e.g., classrooms, homes, work environments), through the concept of triadic reciprocal causality, all of which affect the individual’s functioning (Usher and Schunk, 2018). Based on the above theoretical foundation, a clear causal relationship between environmental factors and personal/behavioral factors was found through our research model, while there were interaction effects between personal and behavioral factors. Adolescents’ environmental factors, especially family environment (parental involvement) and school environment (teacher praise/criticism), will influence adolescents’ collective integration. Poor collective integration will eventually be reflected in adolescents’ behavioral problems and emotional problems.

### Practical implications

In this paper, we applied the cross-lagged model to a large follow-up survey of Chinese adolescents to demonstrate that sleep duration and depression co-occur in adolescence, producing vicious interactions. Besides, this study found that the lasting effects and interactions produced by depression in adolescents are stronger than those produced by sleep duration. Therefore, in order to protect the mental health of adolescents, mediating depression in adolescents takes precedence over extending their sleep duration. This is of particular relevance to parents and schools concerned with adolescents. This study also emphasizes the importance of collective integration of middle school students during adolescence. Also, parents or teachers can have plus or minus effects on students’ group inclusion. For example, parental advice and support as well as teacher praise becomes crucial to collective integration of adolescents.

### Strengths and limitations

The main strength of this study is that it is a large nationally representative survey of adolescents, which allowed us to explore the factors that influence adolescents’ sleep duration and depression. However, this study has several limitations. First, although two waves of CEPS data in this longitudinal study were applied to confirm the enduring interaction between adolescent sleep duration and depression, the time interval between the first and second waves was rather short (1 year). Additional waves could be pursued in the future to elucidate clear long-term effects between adolescent sleep duration and depression. Second, we adopted adolescent self-reported data to measure the variables, which may influence the validity of our findings due to social desirability bias. Future consideration is given to utilizing information from multiple sources, such as parents and teachers.

## Conclusion

In conclusion, this study reports the mediating role of collective integration in the relationships between academic self-efficacy/parental involvement, teacher praise/teacher criticism, and adolescents’ sleep duration and depression by constructing a structural equation model. This study empirically verified that adolescent sleep duration and depression could affect each other and have enduring effects through a two-wave cross-lagged model. In addition, teacher criticism had a greater negative impact on adolescents’ sleep duration and depression than the positive impact of teacher praise. These results suggest that adolescents’ sleep duration in the previous year was a causal predictor of depression in the next year. Similarly, adolescents’ depression in the previous year was a causal predictor of sleep duration in the next year. The lasting effects and interactions produced by depression in adolescents are stronger than those produced by sleep duration. Collective integration was an important mediator of sleep duration and depression, and improving it may be an effective way to increasing sleep duration and reduce the level of depression. Additionally, to prevent sleep deprivation and depression and to improve the collective integration of adolescents, teacher praise needs to be increased and teacher criticism must to be reduced. The predictive power of the model should be further tested using more waves of data. And future research is given to utilizing information from multiple sources in order to avoid social desirability bias, such as parents and teachers.

## Data availability statement

The original contributions presented in the study are included in the article/Supplementary materials, further inquiries can be directed to the corresponding authors.

## Ethics statement

Procedures were approved by the Institutional Review Board in the Renmin University of China. All procedures were performed in accordance with the ethical standards as laid down in the 1964 Declaration of Helsinki and its later amendments or comparable ethical standards. Written informed consent to participate in this study was provided by the participants’ legal guardian/next of kin.

## Author contributions

MG and XL were major contributors in study design, data analysis and interpretation, and drafted the manuscript. C-YL, TC, and Y-CC have made major modifications and improvements to the manuscript. SZ and Y-CC supervised the study and critically reviewed the manuscript several times. HM was contributed in literature review. TC provides funding acquisition. All authors agree to be accountable for all aspects of the work in ensuring that questions related to the accuracy or integrity of any part of the work are appropriately investigated and resolved. All authors read and approved the final manuscript.

## Funding

This paper was funded by the Bill and Melinda Gates Foundation (No: INV-005834), Fundamental Research Funds for the Central Universities (No: 2022-ZY-SX002), and Scientific Research Grant of Fujian Province of China (No. Z0230104). The sponsors of the project had no role in the study design, data collection, data analysis, data interpretation and in writing the manuscript.

## Conflict of interest

The authors declare that they have no known competing financial interests or personal relationships that could have appeared to influence the work reported in this paper.

## Publisher’s note

All claims expressed in this article are solely those of the authors and do not necessarily represent those of their affiliated organizations, or those of the publisher, the editors and the reviewers. Any product that may be evaluated in this article, or claim that may be made by its manufacturer, is not guaranteed or endorsed by the publisher.
