# Supplementary material for: Sleep duration and depression among adolescents: Mediation effect of collective integration
Source: Front Psychol. 2022 Nov 28;13:1015089. doi: 10.3389/fpsyg.2022.1015089 (PMC9744325; doi:10.3389/fpsyg.2022.1015089)
Supplement: Supplementary file 1 [file Data_Sheet_1.docx]

**

**

**Figure 1** Model diagram of the mediating effect of collective integration on sleep duration and depression among adolescents without depression in wave 1.

In this model, sex, live with parents (Yes or No), and parental highest education level are taken as control variables.

AS, Academic self-efficacy; PI, Parental involvement; TP, Teacher praise; TC, Teacher criticism; CI, Collective integration; W1SD, Sleep duration in2013-2014; W1DP, Depression in 2013-2014; W2SD, Sleep duration in 2014-2015; W2DP, Depression in 2014-2015.

^†^p<0.10 (marginally significant); **p* < 0.05, ***p* < 0.01, ****p* < 0.001.

**

**

**Figure 2** Model diagram of the mediating effect of collective integration on sleep duration and depression among adolescents with normal sleep duration in wave 1.

In this model, sex, live with parents (Yes or No), and parental highest education level are taken as control variables.

AS, Academic self-efficacy; PI, Parental involvement; TP, Teacher praise; TC, Teacher criticism; CI, Collective integration; W1SD, Sleep duration in2013-2014; W1DP, Depression in 2013-2014; W2SD, Sleep duration in 2014-2015; W2DP, Depression in 2014-2015.

**p* < 0.05, ***p* < 0.01, ****p* < 0.001.

Table 1 Measures of goodness-of-fit for the competition model among adolescents without depression in wave 1

|  | Chi-square/DF | RMSEA | NFI | TLI | CFI | IFI | CN | GFI | AGFI |
| --- | --- | --- | --- | --- | --- | --- | --- | --- | --- |
| Initial model | 7193.03/550 | 0.040 | 0.93 | 0.93 | 0.94 | 0.94 | 653.78 | 0.98 | 0.98 |
| Delete AS → W1SD | 7182.24/551 | 0.040 | 0.93 | 0.93 | 0.94 | 0.94 | 654.87 | 0.98 | 0.98 |
| ^#^Delete LWP → W1SD | 7192.99/552 | 0.040 | 0.93 | 0.93 | 0.94 | 0.94 | 655.91 | 0.98 | 0.98 |

AS, academic self-efficacy; LWP, live with parents (Yes or No); W1SD, sleep duration in 2013-2014.

^#^ The goodness-of-fit of the Final model

Table 2 Measures of goodness-of-fit for the competition model among adolescents with normal sleep duration in wave 1

|  | Chi-square/DF | RMSEA | NFI | TLI | CFI | IFI | CN | GFI | AGFI |
| --- | --- | --- | --- | --- | --- | --- | --- | --- | --- |
| Initial model | 7229.83/550 | 0.040 | 0.93 | 0.93 | 0.94 | 0.94 | 653.42 | 0.98 | 0.98 |
| Delete AS → W1SD | 7231.06/551 | 0.040 | 0.93 | 0.93 | 0.94 | 0.94 | 654.41 | 0.98 | 0.98 |
| ^#^Delete LWP → W1SD | 7233.58/552 | 0.040 | 0.93 | 0.93 | 0.94 | 0.94 | 655.29 | 0.98 | 0.98 |

AS, academic self-efficacy; LWP, live with parents (Yes or No); W1SD, sleep duration in 2013-2014.

^#^ The goodness-of-fit of the Final model
